# Cerebrolysin Combined with Rehabilitation Enhances Motor Recovery and Prevents Neural Network Degeneration in Ischemic Stroke Patients with Severe Motor Deficits

**DOI:** 10.3390/jpm11060545

**Published:** 2021-06-11

**Authors:** Won Hyuk Chang, Jungsoo Lee, Yong-Il Shin, Myoung-Hwan Ko, Deog Young Kim, Min Kyun Sohn, Jinuk Kim, Yun-Hee Kim

**Affiliations:** 1 Department of Physical and Rehabilitation Medicine, Center for Prevention and Rehabilitation, Heart Vascular and Stroke Institute, Samsung Medical Center, Sungkyunkwan University School of Medicine, Seoul 06351, Korea; wh.chang@samsung.com (W.H.C.); jungsoo0319@gmail.com (J.L.); 2 Department of Rehabilitation Medicine, Pusan National University College of Medicine, Pusan National University Yangsan Hospital, Yangsan 50612, Korea; rmshin01@gmail.com; 3 Department of Physical Medicine and Rehabilitation, Research Institute of Clinical Medicine of Jeonbuk National University, Biomedical Research Institute of Jeonbuk National University Hospital, Jeonju 54907, Korea; mhko@jbnu.ac.kr; 4 Department and Research Institute of Rehabilitation Medicine, Yonsei University College of Medicine, Seoul 03722, Korea; kimdy@yuhs.ac; 5 Department of Rehabilitation Medicine, School of Medicine, Chungnam National University, Daejeon 35015, Korea; mksohn@cnu.ac.kr; 6 Department of Health Sciences and Technology, Department of Medical Device Management & Research, Department of Digital Health, SAIHST, Sungkyunkwan University, Seoul 06351, Korea; longfellow92@naver.com

**Keywords:** stroke, motor recovery, Cerebrolysin, rehabilitation, motor impairment, functional imaging

## Abstract

The objective of this study was to evaluate whether Cerebrolysin combined with rehabilitation therapy supports additional motor recovery in stroke patients with severe motor impairment. This study analyzed the combined data from the two phase IV prospective, multicenter, randomized, double-blind, placebo-controlled trials. Stroke patients were included within seven days after stroke onset and were randomized to receive a 21-day treatment course of either Cerebrolysin or placebo with standardized rehabilitation therapy. Assessments were performed at baseline, immediately after the treatment course, and 90 days after stroke onset. The plasticity of the motor system was assessed by diffusion tensor imaging and resting state fMRI. In total, 110 stroke patients were included for the full analysis set (Cerebrolysin *n* = 59, placebo *n* = 51). Both groups showed significant motor recovery over time. Repeated-measures analysis of varianceshowed a significant interaction between time and type of intervention as measured by the Fugl–Meyer Assessment (*p* < 0.05). The Cerebrolysin group demonstrated less degenerative changes in the major motor-related white matter tracts over time than the placebo group. In conclusion, Cerebrolysin treatment as an add-on to a rehabilitation program is a promising pharmacologic approach that is worth considering in order to enhance motor recovery in ischemic stroke patients with severe motor impairment.

## 1. Introduction

Stroke is a leading cause of adult disability in Korea as well as worldwide [1]. Although stroke mortality rates in Korea have shown substantial declines, the number of patients suffering from residual stroke disability is increasing [2]. Recent advances in diagnoses, management, and rehabilitative treatment have had a significant impact on clinical and functional outcomes after stroke [3]. Even though these advances have had clinical benefits, many stroke survivors still suffer from significant motor impairments [4]. Neuroplasticity is the basic principle for the recovery of motor function after stroke, and strategies to increase neuroplasticity are the gold standard for post-stroke rehabilitation [5]. The subacute stroke stage, characterized by higher neuroplasticity, is a critical period for therapy as, during this stage, the brain is most receptive to modification by rehabilitative experiences [6,7].

Several medications have been reported to improve motor recovery after stroke when used in combination with rehabilitation strategies such as task-specific training [8]. Although there is some evidence that medications may help in enhancing motor recovery after stroke, well-controlled larger trials are needed to confirm that specific medication can facilitate motor recovery after stroke [9]. Whereas many studies have investigated the effects of rehabilitation strategies and pharmacological approaches on motor recovery in stroke patients with moderate to severe motor impairment, few studies have focused on stroke patients with severe motor impairment [10]. Cerebrolysin (EVER Neuro Pharma GmbH, Austria), a neuropeptide preparation of low-molecular-weight neuropeptides (<10 kDa) and free amino acids, has been shown to have neuroprotective and neurorestorative properties by decreasing excitotoxicity, inhibiting free radical formation, activating microglia and apoptosis, exhibiting neurotrophic action, promoting neuronal sprouting, improving cellular survival, and stimulating neurogenesis [11,12]. Meta-analyses showed a beneficial effect of Cerebrolysin on early global neurological deficits and a favorable safety profile in the treatment of acute ischemic stroke [13,14]. Furthermore, in our previous study, we identified an effect of Cerebrolysin on the motor function of stroke patients in the subacute stage of ischemic stroke [15]. There was a significant improvement in motor function in a subgroup of patients with initially severe motor impairment (Fugl–Meyer Assessment (FMA) < 50); however, the sample size in our previous study was not large enough.

We therefore performed a trial with a larger number of participants to clarify the efficacy of Cerebrolysin in promoting additional motor recovery combined with rehabilitation therapy during the subacute phase of stroke in patients with severe motor impairment. In addition, we investigated the effects of Cerebrolysin on neuroplasticity using functional neuroimaging.

## 2. Materials and Methods

### 2.1. Experimental Design

We analyzed data from two phase IV trials (NCT01996761 and NCT02768571) to verify the effect of Cerebrolysin on improving motor function and motor network plasticity in subacute stroke patients with severe motor impairments (FMA < 50) [16]. Patients were included in both studies within the first 7 days after stroke if they suffered from a first cortical or/and subcortical unilateral infarction confirmed by brain CT or MRI, had an inpatient status, and were aged between 18 and 80 years. Exclusion criteria were progressive or unstable stroke, pre-existing and active major neurological disease or major psychiatric disease, a history of significant alcohol or drug abuse within the last 3 years, advanced liver, kidney, cardiac, or pulmonary disease, a terminal medical diagnosis consistent with survival <1 year, pregnancy or lactation, or any condition that contraindicated Cerebrolysin, including allergy to Cerebrolysin. Inclusion and exclusion criteria for the second trial (NCT02768571) were the same as those used in the first trial (NCT02768571) [15], with the exception of motor impairment score measured by a total score of FMA (FMA-T) at the 7th day after stroke onset, which ranged from 0 to 49 [17]. Written informed consent was obtained from all subjects prior to inclusion in the study and study protocols were approved by the Institutional Review Board of each participating center.

Each clinical trial was a prospective, multicenter, randomized, double-blind, placebo-controlled, parallel-group study. The screening visit was performed within seven days after stroke; demographic data, medical history, and data on physical examination and laboratory tests were documented. Enrolled patients were randomized to receive a 21-day treatment course (Days 8–28) of either Cerebrolysin or placebo, given as add-on to standardized rehabilitation therapy. Cerebrolysin was administered once daily at a dosage of 30 mL, diluted with saline (total infusion solution 100 mL), by intravenous infusion over a time period of 30 min. Patients in the control group received daily 100 mL of saline instead. In addition, all patients received a standardized rehabilitation program consisting of two hours of physical therapy and one hour of occupational therapy daily on weekdays (Monday to Friday). Additional language or cognitive rehabilitation sessions were provided as needed. A standardized rehabilitation program was performed according to the clinical practice guidelines for stroke rehabilitation in Korea [18]. After baseline assessment (Day 8; T0), efficacy and safety were assessed immediately after treatment (Day 29; T1) as well as 3 months (Day 90; T2) after stroke onset. Changes in the neuroplasticity of the motor network were assessed by diffusion tensor imaging (DTI) and resting state functional magnetic resonance imaging (rs-fMRI) at T0 and T2. 

### 2.2. Randomization and Blinding

Treatments were assigned according to a predefined randomization plan. A study-specific randomization code was prepared using the SAS^®^ software package (proc plan) in a validated working environment. A block size of 4 was used, and treatment assignment at the ratio of 1:1 was stratified by each clinical center. The size of blocks was not open to the centers. Each center received medication for a sequence of complete blocks so that treatments were balanced within each center. Patients, healthcare providers, data collectors, outcome assessors, and the sponsor were blinded to treatment allocation. The statistician in charge of randomization and the person in charge of preparing the study medication were unblinded.

### 2.3. Functional Assessments

Baseline assessment was performed at T0 using the National Institute of Health Stroke Scale (NIHSS) for stroke severity [19] and the Korean Mini-Mental State Examination (K-MMSE) for cognitive function [20]. Motor function assessments were performed by blinded observers at baseline (T0), immediately after treatment on Day 29 (T1), and at follow-up and 3 months (T2) after stroke onset by using the FMA. We used total FMA scores (FMA-T) as well as the upper (FMA-UL) and lower limb (FMA-LL) scores separately.

In addition, corticospinal tract (CST) integrity at T0 was assessed by measuring the response of the affected motor cortex to transcranial magnetic stimulation (TMS); in particular, motor evoked potentials (MEPs) of the resting paretic first dorsal interosseous (FDI) muscle were measured as described previously [21,22]. MEPs were assessed using a single magnetic stimulation at 120% of the rMT intensity over the ipsilesional M1 using a 70 mm figure-of-eight coil. A Synergy electromyography/evoked potentials system (Medelec, Kingswood, Bristol, UK) was used to record and monitor the activity of the contralateral FDI muscle. Single-pulse TMS was applied over the ipsilesional M1 with a Magstim BiStim2 stimulator (Magstim, Spring Gardens, Wales, UK). The coil was held tangentially to the scalp, with the handle pointing backward and laterally at 45° from the mid-sagittal line. Patients were grouped according to the presence of MEPs on the affected FDI muscle into an “MEP response” group, which included all patients who exhibited MEPs in the affected FDI, and a “no MEP response” group, which included patients who did not exhibit any MEPs with peak-to-peak amplitude of ≥50 µV in the affected FDI according to three successive discharges with maximal stimulator output. Eliciting MEP was a reliable tool for predicting motor recovery in stroke patients [23,24].

### 2.4. Imaging Assessments

Individual stroke lesions were drawn manually by a blinded observer on diffusion-weighted images using FSLview 4.0.1 (part of FSL software version 5.0.9). Each lesion volume was warped to the Montreal Neurological Institute (MNI) standard space using the transformation matrix obtained from each DTI normalization. Results were visualized using MRIcroGL (McCausland Center for Brain Imaging, University of South Carolina, http://www.cabiatl.com/mricrogl, accessed on 7 March 2020). Lesion volumes were flipped for patients with lesions on the left side. All lesions were overlaid on the right side. 

Motor network plasticity was assessed based on DTI and rs-fMRI data. Imaging data were acquired a 3T Philips ACHIEVA^®^ MR scanner (Philips Medical Systems, Best, The Netherlands). All patients were instructed to keep their eyes closed without thinking about anything in particular and to remain motionless during the scan time. In DTI data, 46 whole brain images were acquired. The dataset comprised 45 images with diffusion weighting (b value = 1000 s/mm^2^) applied along 45 diffusion directions, and one image with no diffusion weighting. Acquisition parameters were 60 axial slices, slice thickness = 2.25 mm, no gap, matrix size = 112 × 112, repetition time = 8770 ms, echo time = 60 ms, and field of view = 220 mm × 220 mm. In rs-fMRI data, 100 whole brain images were acquired. Acquisition parameters were 35 axial slices, slice thickness = 4 mm, no gap, matrix size = 128 × 128, repetition time = 3000 ms, echo time = 35 ms, and field of view = 220 mm × 220 mm.

#### 2.4.1. Diffusion Tensor Imaging Data Analysis

Individual DTI data were preprocessed using the FMRIB’s Diffusion Toolbox from FSL software package version 5.0.9 (FMRIB Software Library, FMRIB, Oxford, UK, http://www.fmrib.ox.ac.uk/fsl, accessed on 15 June 2019). Eddy currents and head motion were corrected and brains were skull-stripped. The DTIfit algorithm was used to fit a tensor model and to calculate fractional anisotropy (FA) maps for eddy currents and head motion-corrected data. FA maps were registered non-linearly to the MNI standard space (FMRIB58_FA standard-space image) using the registration algorithm of the tract-based spatial statistics (TBSS) technique. At that time, lesioned voxels were masked out. The stroke lesion was not considered during the registration. Warped FA maps were visually checked. 

To obtain region-specific FA values, the CST template obtained from healthy DTI data and the Johns Hopkins University white matter atlas (JHU ICBM-DTI-81) [25] provided by FSL were used. The following major vertical, longitudinal, lateral, and whole white matter tracts were investigated: the CST, superior corona radiata (SCR), superior longitudinal fasciculus (SLF), whole hemispheric tract (WHT), and corpus callosum. The corpus callosum consists of the genu (GCC, genu of the corpus callosum), body (BCC, body of the corpus callosum), and splenium (SCC, splenium of the corpus callosum). Each region was binarized and masked over the warped FA map. The FA value of a region was obtained by averaging FA values within each region. Proportional FA values (affected/unaffected hemisphere) were calculated for the CST, SCR, SLF, and WHT.

Tract-based spatial statistics (TBSS) were calculated using the FMRIB’s Diffusion Toolbox from FSL software package version 5.0.9 (FMRIB Software Library, FMRIB, Oxford, UK, http://www.fmrib.ox.ac.uk/fsl, accessed on 15 June 2019). Various functions (tbss_1_preproc, tbss_2_reg, tbss_3_postreg, and tbss_4_prestats) were used for non-linear regression. To compare relative voxel-wise FA values between affected and unaffected hemispheres, the tbss_sym function was used.

#### 2.4.2. Resting State Functional Magnetic Resonance Imaging Data Analysis

Preprocessing of rs-fMRI data was performed using the SPM12 package (Welcome Trust Centre for Neuroimaging, University College London, London, UK, http://www.fil.ion.ucl.ac.uk/spm, accessed on 23 May 2019). Preprocessing consisted of several steps: slice timing and head motion correction, registration to structural images, spatial normalization into a template in the MNI atlas space, and spatial smoothing using a 6 mm full width at half-maximum Gaussian kernel. Several nuisance sources were removed using linear regression of nine nuisance parameters, including head motion parameters and temporal parameters of global, white matter, and ventricle signals. Band-pass filtering between 0.009 and 0.08 Hz was performed to remove constant offsets and linear trends. Nuisance regression and band-pass filtering were processed using MATLAB (Mathworks, Natick, MA, USA).

A network consists of a set of nodes and edges between pairs of nodes. In this study, regions of interest (ROIs) were obtained from the previous meta-analyses [26]. We used the “affected upper limb movements vs. rest in stroke patients” results in this study. Twenty-four ROIs were defined as 10-mm-diameter spheres around the predefined MNI coordinates. Lesioned voxels were masked out. The edge of the network was calculated using Pearson’s correlation between the mean time courses of each of the 24 regions. 

Efficiency is a measure of how efficiently information is exchanged. The measure is inversely related to path length. For reference, path length is the minimum number of edges that must be traveled to move from one node to another. The efficiency of a network is defined as follows [27]:

$$E_{global}=\frac{1}{n}\underset{i\in N}{\sum }\frac{\sum_{j\in N,j\neq i}{\left(d_{ij}^{w}\right)}^{-1}}{n-1}$$

where *n* is the number of nodes and $d_{ij}^{w}$ is the shortest weighted path length between node *i* and node *j*.

### 2.5. Statistical Analysis

The full analysis set (FAS) was defined as the population of patients who underwent functional assessments at least at baseline (T0) and immediately after treatment (T1). Missing data were imputed by the last observation carried forward (LOCF) method. The safety population included all patients who received at least one dose of the study medication. SPSS version 24.0 (SPSS, Chicago, IL, USA) was used for statistical analyses. The Shapiro–Wilk normality test was used to determine whether assessment values showed a normal distribution; all assessment values were found to have a normal distribution (*p* > 0.05 by the Shapiro–Wilk normality test). The primary outcome of this study was the pattern of FMA-T from T0 to T2. To test for the effects of Cerebrolysin across all time points (T0, T1, and T2), we used a repeated-measures analysis of variance (ANOVA) with time as the within-patient factor and group (Cerebrolysin vs. placebo) as the between-patient factor. The paired *t*-test was performed to analyze significant changes between time points within groups. In addition, the independent *t*-test was used between the two groups to analyze the change in motor function. To correct for multiple comparisons, the Bonferroni correction was used. The effect of Cerebrolysin or placebo on FMA at T2 and the improvement in FMA from baseline was analyzed by simple linear regression with one independent variable by group. This analysis was performed to evaluate the pattern of motor function improvement at T2 in each group. 

For imaging analysis, the per protocol (PP) set who completed follow-up assessment at T2 was used. The Shapiro–Wilk normality test was used to determine if imaging data were drawn from a normal distribution; all values were found to have a normal distribution (*p* > 0.05 by the Shapiro–Wilk normality test). Repeated-measures ANOVA was used to investigate group and time interaction effects for the imaging measures to investigate motor network plasticity between the two groups. The paired *t*-test was performed to analyze significant changes from T2 to T1 in participants’ imaging measures within groups.

*p*-values less than 0.05 were considered statistically significant.

## 3. Results

### 3.1. Participants

**Figure 1:**
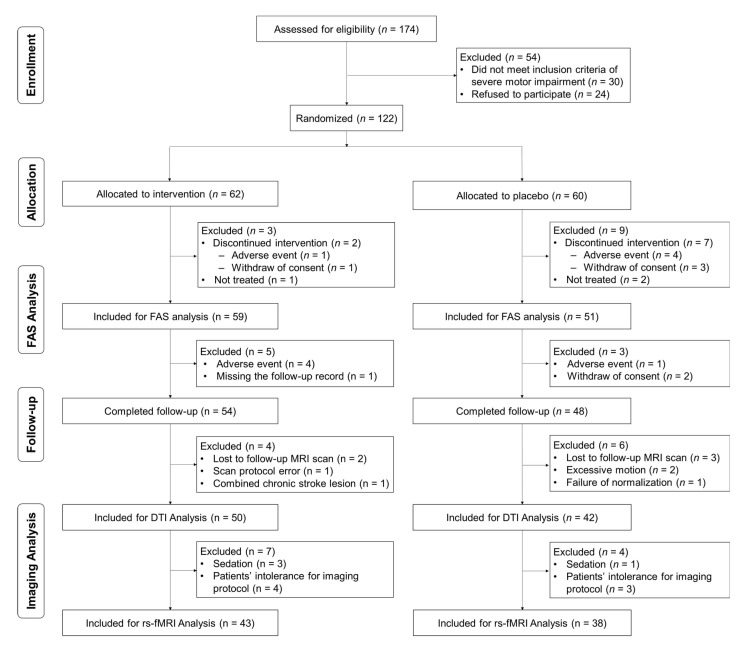
Consort flowchart. FAS, full analysis set; DTI, diffusion tensor imaging; rs-fMRI, resting-state functional MRI.

A total of 174 stroke patients were screened. Of these, 122 patients were randomized after excluding 54 patients who did not meet criteria of severe motor impairment (30 patients) or who refused to participate (24 patients). Three of these (Cerebrolysin *n* = 1, placebo *n* = 2) withdrew their consent before study drug administration. Out of 119 treated patients, nine patients dropped out because of adverse events (*n* = 5) and withdrawal of consent (*n* = 4). Total 110 patients who completed the three-week intervention and underwent functional assessments at least at baseline (T0) and immediately after treatment (T1; Day 29) represent the FAS (Cerebrolysin *n* = 59, placebo *n* = 51) (Figure 1). Forty patients from the first trial dataset (Cerebrolysin *n* = 21, placebo *n* = 19) and 70 patients from the second trial dataset (Cerebrolysin *n* = 38, placebo *n* = 32) were included in the FAS analysis. 

Among the patients who completed follow-up assessments at T2 (Day 90; *n* = 102), structural MRI and DTI data for 10 patients were not included for analysis due to loss to follow-up MRI scan (*n* = 5), a failure of adequate MRI data due to subject motion (*n* = 2), scan protocol error (*n* = 1), a combined chronic stroke lesion (*n* = 1), and a failure of spatial normalization for MRI preprocessing (*n* = 1). Of these, rs-fMRI scans could not be used in 11 patients due to missing data because of patient intolerance for the imaging protocol (*n* = 7) and scans with sedation (*n* = 4). Finally, 92 DTI data (Cerebrolysin *n* = 50, placebo *n* = 42) and 81 rs-fMRI data (Cerebrolysin *n* = 43, placebo *n* = 38) were included for imaging analysis (Figure 1). 

**Figure 2:**
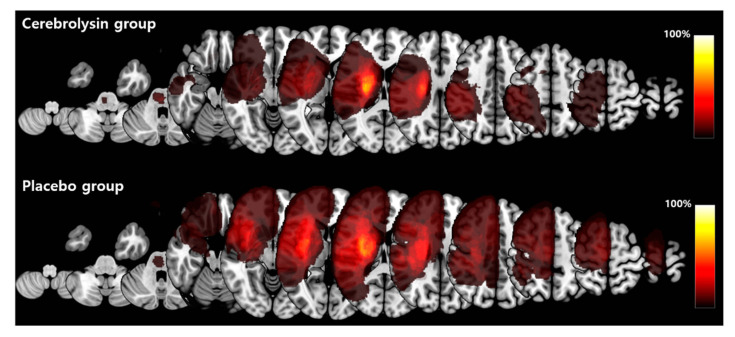
Group comparison of lesion maps. Each patient’s lesion volume was fitted to the Montreal Neurological Institute (MNI) standard space and results were visualized using MRIcroGL (McCausland Center for Brain Imaging, University of South Carolina, http://www.cabiatl.com/mricrogl, accessed on 7 March 2020). For group comparison, stroke lesions on the left side were flipped to be overlaid on the right hemisphere. The colored bar indicates the percentage of lesion areas in each group.

Table 1 summarizes the characteristics of the total 110 patients included in the FAS analysis. There were no significant differences in demographic and clinical characteristics between the two groups, including initial stroke severity measured by NIHSS, motor impairments, and lesion volume. Lesion maps showed a similar pattern in both groups (Figure 2). 

### 3.2. Motor Function Outcomes

**Figure 3:**
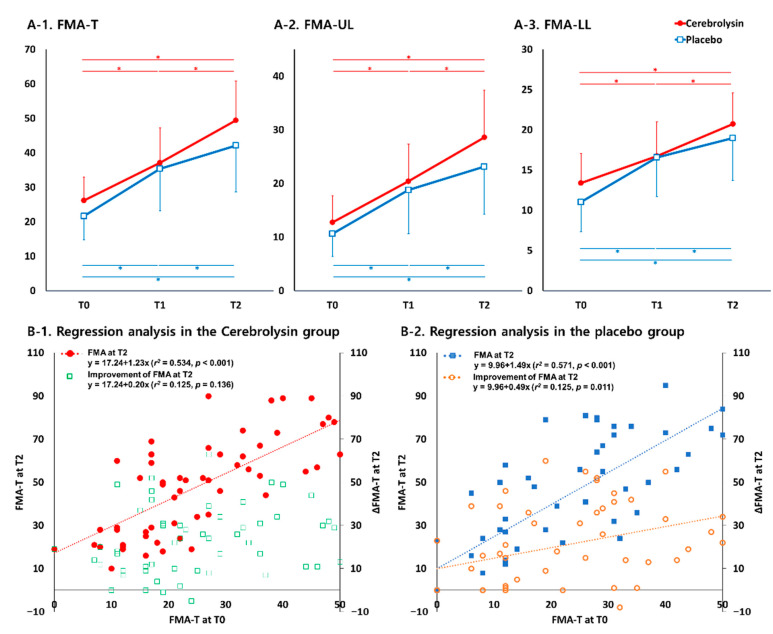
Changes in Fugl-Meyer assessment (FMA) in the Cerebrolysin and placebo groups. (**A-1**). Time courses for total FMA; (**A-2**). Time course for upper limb of FMA (FMA-UL); (**A-3**). Time course for lower limb of FMA (FMA-LL); A simple regression analysis of the effect of FMA-T baseline scores (T0) on FMA-T at Day 90 (T2) and the improvement from baseline (T0) is shown for Cerebrolysin (**B-1**) and placebo (**B-2**) groups. * *p* < 0.05 between time points in each group (repeated-measures ANOVA).

Figure 3 shows the change in motor function for each group. Each group improved significantly over time in the FMA-T (*p* < 0.05), and repeated-measures ANOVA showed a significant interaction effect between time and type of intervention in the FMA-T (F2,107 = 3.202, *p* = 0.045, Figure 3(A-1). The improvements from T0 to T1 and T0 to T2 did not show significant group differences; however, the improvement from T1 to T2 was significantly larger in the Cerebrolysin group than the placebo group (*p* < 0.05 and Cohen’s d =0.47, Table 2).

Both groups showed a significant improvement over time on FMA-UL and FMA-LL scores (*p* < 0.05). Repeated-measures ANOVA demonstrated an interaction effect between time and type of intervention in both the upper (FMA-UL) and lower (FMA-LL) limbs (F2,107 = 2.635, *p* = 0.076 and F2,107 = 2.825, *p* = 0.064, Figure 3(A-2,A-3)). The change in the FMA-UL scores from T1 to T2 was significantly larger in the Cerebrolysin group than the placebo group (*p* < 0.05 and Cohen’s d = 0.44; Table 2).

Simple linear regression showed a significant relationship between FMA scores at T0 and T2, both in the Cerebrolysin (r^2^ = 0.534, *p* < 0.001) and the placebo groups (r^2^ = 0.571, *p* < 0.001) (Figure 3(B-1)). In terms of improvement from T0 to T2, a significant relationship with FMA score at T0 was observed only in the placebo group (r^2^ = 0.125, *p* = 0.011, Figure 3(B-2)), and not in the Cerebrolysin group. These findings suggest that there was an unexpected improvement in motor function at T2 in patients treated with Cerebrolysin when compared to the placebo group. 

For both upper and lower limbs scores, simple linear regression showed a significant relationship between T0 and T2, for both Cerebrolysin (UL: r^2^ = 0.504, *p* < 0.001; LL: r^2^ = 0.428, *p* < 0.001) and placebo (UL: r^2^ = 0.571, *p* < 0.001; LL: r^2^ = 0.527, *p* < 0.001). In terms of improvement from T0 to T2, a significant relationship with FMA-UL at T0 was observed in the placebo group (r^2^ = 0.101, *p* = 0.023), but not in the Cerebrolysin group for FMA-UL. These findings mean that a distinctive outcome was observed in the Cerebrolysin group with respect to improvement of upper extremity motor function at T2.

In the MEP study, 20 (33.9%) out of 59 patients showed a positive MEP response at T1 in the Cerebrolysin group and 14 (27.5%) out of 51 patients in the placebo group. The MEP response rates were significantly increased at T2 in both groups (*p* < 0.05); 42.4% (25 out of 59 patients) in the Cerebrolysin group and 35.3% (18 out of 52 patients) in the placebo group. However, there was no significant group difference in MEP response rate between the Cerebrolysin and placebo groups at T1 and T2.

### 3.3. Results of Functional Imaging Data Analysis

Table 3 shows the results of DTI data analyses. There was no significant difference in FA values at T0 between the two groups. In each group, there was a significant decrease from T0 to T2 in proportional FA values of CST, SCR, SLF, and WHT (*p* < 0.05). However, repeated-measures ANOVA showed a significant interaction between time and type of intervention as measured by proportional FA values of CST, SCR, SLF, and WHT (*p* < 0.05). These FA values were more decreased in the placebo group compared to the Cerebrolysin group. Corpus callosum FA values showed the same trends, with changes in major vertical and longitudinal white matter tracts in each group. FA values decreased significantly in each group from T0 to T2 (*p* < 0.05). However, repeated-measures ANOVA showed a significant interaction between time and type of intervention as measured by FA values of the GCC, BCC, and SCC (*p* < 0.05). These FA values were more decreased in the placebo group compared to the Cerebrolysin group.

**Figure 4:**
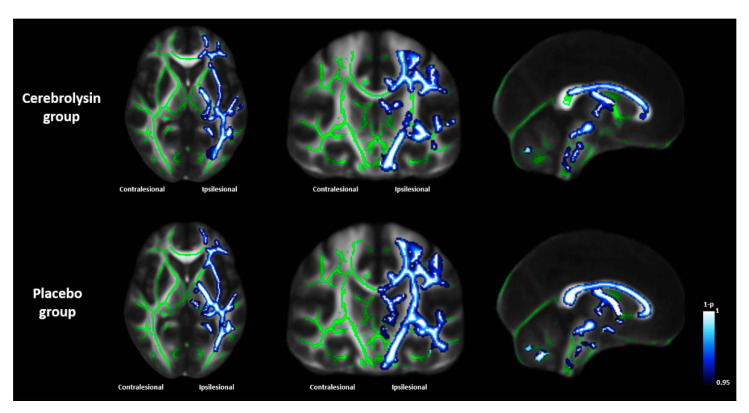
Changes in voxel-wise fractional anisotropy values from baseline (T0) to Day 90 (T2) using the tract-based spatial statistics technique. The results are presented in axial (z = 3), coronal (y = -21), and sagittal (x = 0) views in MNI standard space. Green voxels represent the mean white matter skeleton of all subjects. Blue-white voxels represent significantly decreased fractional anisotropy of white matter tracts in each group (*p* < 0.05).

Figure 4 shows TBSS results for the axial, coronal, and sagittal views in each group. Changes in voxel-wise FA values from T0 to T2 showed different patterns between the Cerebrolysin and placebo groups. In the Cerebrolysin group, white matter areas showing a significant FA decrease were smaller than in the placebo group (*p* < 0.05). In particular, differences were noticeable in the internal capsule in the axial view, CST in the coronal view, and corpus callosum in the sagittal view. These TBSS results were consistent with the FA analyses based on DTI.

Rs-fMRI data analysis showed no significant difference in examined connectivities at T0 between the two groups. In the Cerebrolysin group, there was no significant change from T0 to T2 in ipsilesional, contralesional, or interhemispheric connectivities and network efficiency; however, there were significant decreases from baseline T0 to T2 in ipsilesional connectivity and network efficiency in the placebo group (*p* < 0.05). In addition, repeated-measures ANOVA showed a significant interaction between time and type of intervention as measured by ipsilesional connectivity (*p* < 0.05, Table 3). These results suggest that the motor network of the affected hemisphere was better preserved in the Cerebrolysin group than the control group.

### 3.4. Safety Analysis

Of all patients treated with the studied medication (*n* = 119), a total of 92.4% received 21 infusions (Cerebrolysin 96.7%, placebo 87.9%). Although five patients in the Cerebrolysin group (8.1%) and five in the placebo group (8.3%) suffered from an adverse event, none of the adverse events were related to the studied medication. During the intervention period, one patient in the Cerebrolysin group dropped out due to stroke progression and four patients in the placebo group due to a hemorrhagic transformation of cerebral infarction (1), motor weakness (1), stroke progression (1), and stroke recurrence (1). All five patients recovered during the study period. In the post-intervention period, four patients in the Cerebrolysin group were lost to follow-up due to stroke recurrence (1), rectal hemorrhage (1), and pneumonia (2). Two of these patients recovered during the study period, one patient with stroke recurrence showed aggravation of neurologic symptoms, and one patient with aspiration pneumonia died. One patient in the placebo group was lost to follow-up due to stroke recurrence, which resolved during the study period. Vital signs and laboratory values were similar between the two groups and showed no clinically relevant changes during the course of the study.

## 4. Discussion

This study showed that a three-week conventional rehabilitation therapy in combination with Cerebrolysin provides additional benefits to conventional rehabilitation therapy alone in terms of motor recovery in patients with severe motor impairment. 

A possible effect by which Cerebrolysin promotes motor recovery may be the preservation of the motor network in stroke patients. To promote neurologic motor recovery of impaired limbs, new therapeutic strategies, including non-invasive brain stimulation and robotic therapies, have been used as additional therapies to complement conventional rehabilitation strategies, such as task-specific training, electromyographic biofeedback, functional electrical stimulation, and constraint-induced movement therapy; however, motor recovery in some stroke survivors is still not satisfactory [28]. In addition, there was a lack of clear evidence that new therapeutic strategies tested in large multicenter trials were superior to conventional stroke rehabilitation [29]. New therapeutic strategies that focus on promoting neuroplasticity are being developed to enhance motor recovery after stroke [30]. Many pharmacotherapy trials have also been conducted, because drugs administered to stroke patients may influence neuroplasticity [10]. Selective serotonin reuptake inhibitors (SSRIs) are currently widely used in stroke patients due to their ability to promote neuroplasticity and enhance motor recovery [31]. SSRIs are thought to exert these effects by modulating inhibitory pathways, thereby enhancing reorganization and reestablishing excitatory–inhibitory control. These inhibitory-modulating effects may play a key role in learning-induced plasticity in neural circuits in stroke patients [31]. Several trials of dopamine agonists have reported that these agonists promote motor recovery after stroke [10]. The potential mechanisms of the dopamine-mediated improvement in motor recovery are the potentiation of drive and arousal in conditioned learning and the up-regulation of glutaminergic transmission, which modulates synaptic efficacy [32]. Nevertheless, the clinical effects of pharmacotherapy are limited in stroke patients with severe motor impairment because current motor rehabilitation strategies are focused on the reorganization of preserved motor networks after stroke [33]. Stroke patients with severe motor impairment have relatively larger lesions and lower preservation of motor networks than those with mild motor impairment. Novel pharmacotherapies with neuroprotective as well as neurotrophic action are needed to promote motor recovery. Cerebrolysin (EVER Neuro Pharma GmbH, Unterach am Attersee, Austria, 30 mL infusion), a unique neuropeptide preparation of low-molecular-weight neuropeptides (<10 kDa) and free amino acids, has been shown to have neuroprotective and neurorestorative properties [11,12]. Cerebrolysin has been reported to increase neuroprotective effects by protecting against excitotoxicity and oxidative stress as well as modulating the inflammatory response [34]. In addition, Cerebrolysin could enhances neurogenesis and neurorestoration through the activity of the neurotrophic factor and sonic hedgehog signaling pathways [35]. A meta-analysis of nine randomized trials reported that Cerebrolysin showed beneficial effects on both neurological deficits and functional outcomes even when its administration was initiated within three days after stroke onset [13]. The recent review article revealed that Cerebrolysin could play a major role in the treatment of many neurological diseases, such as stroke, neurodegeneration, and traumatic brain injury, although more robust clinical data are needed to clarify the effects of Cerebrolysin [35]. Based on animal and human studies, it has been hypothesized that the subacute stroke phase constitutes an interval of heightened plasticity in which neural reorganization occurs, suggesting the existence of a critical period for motor recovery [36]. In this regard, Cerebrolysin administration combined with rehabilitation during the subacute phase may be effective at promoting motor recovery in stroke patients with severe motor impairment.

There was a significant difference in motor recovery pattern between the Cerebrolysin and placebo groups in this study. Enhancement of motor function was observed mainly in the upper extremities rather than in the lower extremities after the 21-day treatment course. Although the motor recovery pattern was similar during the 21-day treatment course, there was significantly greater motor recovery in the Cerebrolysin group from completion of the 21-day treatment to three months after stroke than in the placebo group. These late effects of Cerebrolysin might be due to the characteristics of the participants, who had severe motor impairment at baseline. In contrast to stroke patients with mild motor impairment, stroke patients with severe motor impairment continue to recover motor function from 30 days to 90 days [37]. In addition, functional recovery refers to enhanced sensory and motor performance by stroke patients, and this performance could be followed by pure recovery, which is based on remapping between related cortical regions in order to form new structural and functional circuits [38]. After the hyperacute period, spontaneous repair-related events occur during a period of several weeks in the brains of stroke patients [33]. In the present study, Cerebrolysin was administered during this period, as this is the most important therapeutic time window for functional recovery [39]. The strongest predictor of motor outcome at 3 months in stroke patients is motor function in the acute phase. In addition, motor recovery is known to be correlated with motor function at the acute phase [40,41]. The results of simple regression analysis showed that the improvement in motor function measured by FMA-T and FMA-UL was independent of baseline severity in the Cerebrolysin group. Consistent with this, there was also more pronounced motor improvement in the Cerebrolysin group than in the placebo group in stroke patients with severe motor impairment. 

Motor network plasticity was investigated by functional neuroimaging with DTI and rs-fMRI to determine the effects of Cerebrolysin on neuroplasticity. DTI analysis showed significant interactions between intervention type and time on major vertical, longitudinal, and lateral white matter integrity. Major white matter tracts showed better preservation of integrity in the Cerebrolysin group than the placebo group, as measured by changes in FA values. In addition, TBSS results based on DTI analysis demonstrated less of a decrease in integrity of white matter in the Cerebrolysin group than the placebo group. FA maps from DTI are frequently used to investigate the degree of damage of white matter and recovery in white matter integrity after stroke because these maps are highly sensitive to microstructural changes [42,43]. The FA value of a damaged tract decreases from the acute to the chronic stage of stroke due to Wallerian degeneration [44,45]. TBSS, a technique that combines the strengths of both voxel-wise and tractography-based analyses [46], found that Cerebrolysin protected white matter tracts during the subacute stroke phase. Rs-fMRI analysis showed significant interactions between intervention type and time in ipsilesional functional connectivity, with preservation until Day 90 in the Cerebrolysin group only. These results suggest that the recovery of motor function was due to preservation of the motor network, which was more pronounced in the Cerebrolysin group than the placebo group.

This study has some limitations. First, there was no standardized training program for all physical and occupational therapists in the five hospitals that participated in this study. To minimize this bias, group allocation was performed and balanced in each institute and all institutions provided a standard rehabilitation program according to the clinical guidelines used in Korea. In addition, the actual amount of rehabilitation during the study period was not measured in each participant. Because there was no difference in the incidence of side effects between the two groups, the general patient condition could be similar between the two groups. However, this insufficient information would be one of the limitations in this study.

A recent meta-analysis [47] reported that the use of fluoxetine showed no significant effect on motor recovery in stroke patients, although some studies showed that fluoxetine could substantially enhance upper and lower limb motor recovery in stroke patients. A recent study with a mouse photothrombotic model showed that a combination of fluoxetine with physical rehabilitation was effective for motor recovery in mice after stroke [48]. Therefore, future studies would be beneficial where fluoxetine can be combined with different types and amounts of rehabilitation therapy for motor recovery in stroke patients. In general, the manuscript provides an overly positive impression of the use of pharmacotherapies in stroke rehabilitation; however, there is no definitive support for their use in routine clinical care. Some factors, such as baseline motor severity [15] and combination with rehabilitation [49], should be considered when prescribing Cerebrolysin to stroke patients. Therefore, pharmacotherapies including Cerebrolysin should not be routinely prescribed in stroke rehabilitation.

## 5. Conclusions

We showed that Cerebrolysin had a positive influence on motor network plasticity and a beneficial effect on motor recovery in stroke patients with severe motor impairment. Cerebrolysin treatment for patients in the subacute stroke phase could have important implications for stroke rehabilitation, because conventional rehabilitation strategies appear to have limited ability to promote motor recovery in stroke patients with severe motor involvement [28]. Administration of Cerebrolysin over three weeks in combination with rehabilitation therapy in the subacute stroke phase was safe and well-tolerated. Our study results suggest that Cerebrolysin should be considered in addition to conventional therapy to improve motor recovery in ischemic stroke patients with severe motor impairment.

## Figures and Tables

**Table 1:** Comparison for baseline characteristics of participants.

Parameters	Cerebrolysin (*n* = 59)	Placebo (*n* = 51)	*p*-Value
Sex (male:female)	34:25	32:19	0.585
Age (mean ± SD, years)	68.6 ± 10.8	66.1 ± 12.8	0.246
Stroke side (Rt:Lt)	21:38	26:25	0.104
Baseline stroke severity (mean ± SD)			
NIHSS	9.0 ± 3.9	9.0 ± 4.0	0.882
MMSE	19.6 ± 9.0	18.7 ± 10.1	0.618
FMA-T	26.2 ± 13.6	21.6 ± 13.7	0.085
FMA-UL	12.8 ± 9.9	10.6 ± 8.4	0.220
FMA-LL	13.4 ± 7.4	11.0 ± 7.4	0.097
Lesion volume (cm^3^)	43.9 ± 66.7	69.9 ± 90.5	0.117
MEP response (*n* (%))	13 (22.0%)	10 (19.6%)	0.817

NIHSS, National Institutes of Health Stroke Scale; MMSE, Mini-Mental State Examination; FMA-T, total Fugl–Meyer assessment; FMA-UL, upper limb of Fugl–Meyer assessment; FMA-LL, lower limb of Fugl–Meyer assessment; MEP, motor evoked potential.

**Table 2:** Changes in Fugl–Meyer Assessment scores over time in the Cerebrolysin and placebo groups (full analysis set).

Parameter		T0–T1	T1–T2	T0–T2
ΔFMA-T	Cerebrolysin	11.0 ± 11.8	12.3 ± 13.4 *	23.3 ± 15.9
	Placebo	13.8 ± 15.2	6.7 ± 10.5	20.5 ± 18.8
ΔFMA-UL	Cerebrolysin	7.6 ± 8.7	8.2 ± 9.3 *	15.8 ± 12.6
	Placebo	8.2 ± 11.0	4.4 ± 8.1	12.5 ± 13.3
ΔFMA-LL	Cerebrolysin	3.3 ± 5.0	4.0 ± 6.0	7.4 ± 6.3
	Placebo	5.6 ± 6.0	2.4 ± 4.8	8.0 ± 7.2

Values are means ± SDs; FMA-T, total Fugl–Meyer Assessment; FMA-UL, upper limb of FMA; FMA-LL, lower limb of FMA; * *p* < 0.05 compared with the placebo group.

**Table 3:** Changes in functional neuroimaging parameters over time in the Cerebrolysin and placebo groups.

	Cerebrolysin Group			Placebo Group			*p*-Value
	T0	T2	*p*-Value	T0	T2	*p*-Value	
**Diffusion tensor imaging**							
Corticospinal tract	0.901 ± 0.070	0.816 ± 0.095	<0.001 *	0.885 ± 0.084	0.757 ± 0.113	<0.001 *	0.0167 ^†^
Superior corona radiata	0.887 ± 0.103	0.833 ± 0.145	<0.001 *	0.859 ± 0.115	0.747 ± 0.175	<0.001 *	0.0185 ^†^
Superior longitudinal fasciculus	0.917 ± 0.145	0.861 ± 0.199	<0.001 *	0.908 ± 0.127	0.800 ± 0.225	<0.001 *	0.0305 ^†^
Whole hemispheric tracts	0.935 ± 0.063	0.897 ± 0.082	<0.001 *	0.925 ± 0.064	0.856 ± 0.110	<0.001 *	0.0263 ^†^
Genu of corpus callosum	0.480 ± 0.046	0.465 ± 0.050	<0.001 *	0.485 ± 0.039	0.455 ± 0.056	<0.001 *	0.0091 ^†^
Body of corpus callosum	0.516 ± 0.054	0.494 ± 0.056	<0.001 *	0.523 ± 0.042	0.486 ± 0.062	<0.001 *	0.0256 ^†^
Splenium of corpus callosum	0.629 ± 0.059	0.612 ± 0.061	<0.001 *	0.638 ± 0.044	0.611 ± 0.058	<0.001 *	0.0449 ^†^
**Resting-state functional imaging**							
Ipsilesional connectivity	0.222 ± 0.088	0.217 ± 0.079	0.6735	0.238 ± 0.065	0.191 ± 0.078	0.0014 *	0.0280 ^†^
Contralesional connectivity	0.295 ± 0.084	0.270 ± 0.074	0.0741	0.292 ± 0.059	0.281 ± 0.086	0.4588	0.4866
Interhemispheric connectivity	0.436 ± 0.151	0.448 ± 0.137	0.5095	0.403 ± 0.151	0.415 ± 0.151	0.5825	0.9731
Network efficiency	0.313 ± 0.057	0.302 ± 0.060	0.2386	0.314 ± 0.044	0.294 ± 0.058	0.0270 *	0.4600

* *p* < 0.05, Comparison with value at T0; ^†^ *p* < 0.05, Interaction between time and type of intervention; T0, Baseline assessment at 8 days after stroke onset; T1, After 21 days of Cerebrolysin or placebo intervention; T2, 90 days after stroke onset; Diffusion tensor imaging (Cerebrolysin *n* = 50, Placebo *n* = 42), Resting-state functional magnetic resonance imaging (Cerebrolysin *n* = 43, Placebo *n* = 38.

## Data Availability

The data that support the findings of this study are available from the corresponding author upon reasonable request.
